# Association between plasma transfusion and in-hospital mortality in severe pneumonia: a propensity score-matched retrospective cohort study

**DOI:** 10.1186/s12890-026-04497-4

**Published:** 2026-07-18

**Authors:** Linyan Wu, Zekui Fang, Xiping Wu, Kunzuo Yu, Ning Jiang, Minhong Su

**Affiliations:** 1 Department of Transfusion Medicine, Zhujiang Hospital, Southern Medical University, Guangzhou, Guangdong China; 2 Department of Pulmonary and Critical Care Medicine, Zhujiang Hospital, Southern Medical University, Haizhu District, 253 Gongye Middle Avenue, Guangzhou, Guangdong China; 3 Department of Transfusion, Huidong People’s Hospital, Huizhou, Guangdong China; 4 Department of Urology, Zhujiang Hospital, Southern Medical University, Haizhu District, 253 Gongye Middle Avenue, Guangzhou, China

**Keywords:** Plasma transfusion, Severe pneumonia, Propensity score matching, Mortality, Retrospective cohort study, Confounding by indication

## Abstract

**Background:**

Plasma transfusion is used in patients with severe pneumonia who develop coagulopathy or bleeding, but recipients are often sicker than non-recipients. We evaluated the association between plasma transfusion and in-hospital mortality after adjustment for available measured confounders, while recognizing that unmeasured confounding from transfusion indications and illness severity may persist.

**Methods:**

This single-center retrospective cohort included 156 adults with severe pneumonia admitted between January 2021 and May 2024; 62 received plasma and 94 did not. The matched mortality comparison using propensity score matching (PSM) and McNemar analysis was prespecified as the primary analysis. Supportive and exploratory analyses included Cox regression, Kaplan–Meier analysis, IPTW, overlap weighting, Fine-Gray modeling, E-value analysis, and exploratory coagulation-enhanced, logistic, microbiological/coagulation, and time-dependent exposure sensitivity analyses.

**Results:**

Before matching, mortality was higher among plasma recipients than controls (64.5% vs. 28.0%; crude odds ratio [OR] 4.69, 95% confidence interval [CI] 2.35–9.34; *p* < 0.001), with substantial baseline imbalance. After matching, mortality remained numerically higher in the plasma group (50.0% vs. 36.1%) but was not statistically significant (matched OR 1.62, 95% CI 0.67–3.92; McNemar *p* = 0.383). Cox models yielded attenuated HRs after adjustment, but the fully adjusted model violated the global proportional hazards assumption; therefore, Cox estimates were interpreted as time-weighted associations over follow-up rather than constant effects. Exploratory sensitivity estimates varied in direction across estimands: coagulation-enhanced matching yielded OR 1.50 (95% CI 0.53–4.21), full-cohort logistic regression yielded adjusted OR 3.19 (95% CI 1.12–9.11), approximate adjusted time-dependent Cox analysis yielded HR 1.73 (95% CI 0.85–3.51), and strict verified-date adjusted time-dependent Cox analysis yielded HR 2.67 (95% CI 1.38–5.16).

**Conclusions:**

The attenuation of the crude mortality difference after propensity score matching is consistent with baseline severity and confounding by indication accounting for a substantial component of the unadjusted association; however, residual confounding cannot be excluded. Because residual confounding, immortal-time bias, related time-dependent exposure bias, and proportional-hazards violations remain important limitations, these data do not establish a definitive beneficial or harmful effect of plasma transfusion. The non-significant matched result reflects limited statistical power (approximately 25% for a paired OR of 2.0) and should not be interpreted as evidence of no effect.

**Supplementary Information:**

The online version contains supplementary material available at https://doi.org/10.1186/s12890-026-04497-4.

## Background

Severe pneumonia remains a leading cause of morbidity and mortality worldwide, with in-hospital mortality rates ranging from 20 to 50% depending on disease severity and patient characteristics [1–4]. For the clinician managing these patients, a recurrent and difficult question arises: when a patient with severe pneumonia develops laboratory coagulopathy, clinical bleeding, or progressive deterioration, should plasma be transfused?

The rationale for plasma transfusion in this setting is physiologically straightforward. Severe infection can deplete or consume coagulation factors, and plasma provides a source of replacement factors that might support hemostasis and possibly modulate the inflammatory response. Plasma is widely available in most hospitals, and the decision to transfuse is often made on clinical judgment alone, in the absence of trial evidence or guideline recommendations specific to severe pneumonia, although general evidence-based guidance exists for broader plasma transfusion indications [5]. Yet the net clinical effect of this common practice remains unknown.

The existing evidence is mixed and does not provide clear guidance at the bedside. Several observational studies have reported that plasma transfusion is associated with worse outcomes in critically ill patients, including increased mortality, transfusion-related acute lung injury (TRALI), nosocomial infection, and circulatory overload [6, 7]. A randomized trial of prophylactic plasma transfusion before invasive procedures in critically ill patients with coagulopathy found no reduction in bleeding complications [8]. Conversely, other investigators have suggested that plasma products may have immunomodulatory or endothelial-stabilizing effects that could benefit patients with severe infections [9, 10]. The clinician is left with uncertainty: the crude observational data appear to signal harm, but the patients who receive plasma are visibly sicker, and it is unclear whether the mortality difference reflects a transfusion effect or the underlying illness that prompted the transfusion.

A fundamental challenge in interpreting observational data on plasma transfusion is confounding by indication: patients who receive plasma are systematically sicker than those who do not, creating a potentially spurious association between plasma and adverse outcomes [11]. In unadjusted comparisons in the present cohort, plasma recipients had lower hemoglobin, lower platelet counts, prolonged activated partial thromboplastin time (APTT), and a higher rate of invasive mechanical ventilation compared with controls, all indicating more severe baseline illness. Without careful adjustment for these available severity markers, any observed mortality difference is likely confounded by the illness severity and clinical indications that prompted transfusion.

Propensity score matching (PSM) provides an analytical approach for adjusting for available measured confounders in observational studies by creating more comparable groups for analysis [12]. When combined with complementary analyses, PSM can help evaluate how much an unadjusted association depends on measured baseline imbalance, while still leaving open the possibility of residual confounding from unmeasured factors.

The primary aim of this study was to evaluate whether plasma transfusion is associated with higher in-hospital mortality in patients with severe pneumonia after adjustment for available measured confounders. The matched mortality comparison after PSM was defined as the primary analysis; time-to-event, weighted, competing-risk, logistic, coagulation-enhanced, and timing analyses were considered supportive or exploratory sensitivity analyses intended to characterize uncertainty and estimand dependence.

## Methods

### Study design and setting

This was a single-center retrospective cohort study conducted at Zhujiang Hospital, Southern Medical University, Guangzhou, China. The study period was from January 2021 to May 2024. The study was approved by the Medical Ethics Committee of Zhujiang Hospital, Southern Medical University (approval no. 2024-KY-036–01; approval document dated 2024–02–21). The requirement for informed consent to participate was waived by the Medical Ethics Committee of Zhujiang Hospital, Southern Medical University, as this was a retrospective study using de-identified clinical data. The study was conducted in accordance with the Declaration of Helsinki and reported in accordance with the Strengthening the Reporting of Observational Studies in Epidemiology (STROBE) guidance [13].

### Study population

Patients were eligible for inclusion if they were aged > = 18 years and diagnosed with severe pneumonia according to the 2007 Infectious Diseases Society of America/American Thoracic Society (IDSA/ATS) consensus criteria [14], defined as meeting at least one major criterion (requirement for vasopressors or invasive mechanical ventilation) or three or more minor criteria (respiratory rate > = 30 breaths/min, ratio of partial pressure of arterial oxygen to fraction of inspired oxygen [PaO2/FiO2 ratio] < = 250, multilobar infiltrates, confusion, uremia, leukopenia, thrombocytopenia, hypothermia, or hypotension requiring aggressive fluid resuscitation). Structured fields for vasopressor use, minor-criteria counts, and classification as community-acquired, hospital-acquired, or ventilator-associated pneumonia were not available.

Patients were divided into two groups based on whether they received plasma transfusion during hospitalization: the plasma group (*n* = 62) and the control group (*n* = 94). Plasma transfusion was administered at the discretion of the treating physicians based on clinical judgment. In local practice, plasma may be used for active bleeding, peri-procedural correction, coagulopathy such as prolonged PT or APTT, DIC, or empirical supportive therapy during clinical deterioration; however, these indications were not consistently captured as structured, analyzable patient-level variables.

### Data collection

Clinical data were extracted from electronic medical records and included: demographic characteristics (age, sex); laboratory values at admission (hemoglobin, platelet count, white blood cell count, neutrophil count, lymphocyte count, C-reactive protein [CRP], procalcitonin, alanine aminotransferase [ALT], aspartate aminotransferase [AST], creatinine, PT, APTT, fibrinogen, lactate dehydrogenase); disease severity indicators (PaO2/FiO2 ratio, invasive and non-invasive ventilation status); available comorbidity-related fields in the cleaned analytic dataset (hypertension, dyslipidemia, diabetes mellitus, chronic pulmonary disease, chronic kidney disease, chronic liver disease, cardiovascular/coronary heart disease, cerebrovascular disease, malignancy, prior renal transplantation, and immunosuppressant use); DIC as a separate acute coagulation-related record field; the source-dataset aggregate comorbidity-count variable; coronavirus disease 2019 (COVID-19) infection status; and outcomes (in-hospital mortality, hospital length of stay). The source dataset provided the aggregate 'number of comorbidities' variable without a documented derivation rule; therefore, its exact per-patient composition could not be fully reconstructed from the available source files and cleaned fields. Sequential Organ Failure Assessment (SOFA) score was unavailable in the analyzable dataset and therefore could not be included in the adjustment models.

For the plasma group, additional data on plasma transfusion were collected, including total volume, duration of use, and onset day of plasma initiation where available. Total plasma volume was available for 60 of 62 plasma recipients and duration of plasma use for all 62 plasma recipients; however, body weight was available for only 19 plasma recipients, so reliable weight-based dose analyses in mL/kg could not be performed. The available records did not contain consistently structured, analyzable fields distinguishing ordinary frozen plasma from fresh frozen plasma (FFP). Therefore, the exposure was defined as any plasma transfusion during hospitalization, and subtype-specific analyses could not be performed. Structured data on the precise clinical indication for each plasma transfusion episode were not consistently available. Although source records contained onset-day information for plasma initiation, exact admission-relative initiation time was not consistently reliable enough for a prespecified primary time-dependent or landmark analysis.

### Outcome definitions

The primary outcome was in-hospital mortality. Patients discharged against medical advice with documented terminal prognosis were classified as deaths in the primary analysis, consistent with a conservative worst-case approach. Patients transferred to another hospital or discharged alive were treated as non-deaths for the binary outcome and censored at the transfer or discharge date in Kaplan–Meier and Cox analyses. One control patient had missing mortality status and was excluded from mortality proportion and outcome-model calculations. A sensitivity analysis was performed classifying patients discharged against medical advice as survivors.

The secondary outcome was hospital length of stay, calculated from admission to discharge or death.

### Propensity score matching

Propensity scores were estimated using logistic regression with plasma transfusion as the dependent variable and the following covariates as predictors: age, sex, PaO2/FiO2 ratio, hemoglobin, platelet count, APTT, invasive ventilation status, and recorded comorbidity count. The recorded comorbidity count used in the primary propensity score model was the pre-existing source-dataset aggregate 'number of comorbidities' variable, entered as originally recorded. Because the source file did not provide a documented derivation rule, the individual components of this aggregate count could not be reconstructed with complete certainty. DIC was captured separately as an acute coagulation-related record field, was sparse (3/156 patients, all in the plasma group), and was not entered as an independent covariate in the primary propensity score model. In a sensitivity analysis, redefining the comorbidity count as the explicit sum of the available chronic/immune-status fields with DIC excluded produced a materially unchanged primary matched estimate (matched OR approximately 1.4 versus 1.6; both non-significant), indicating that the primary result did not depend on whether DIC was incorporated into the source aggregate count. Platelet count was included in the propensity score model to improve exposure prediction but was not included in the Cox regression models to limit overfitting in the complete-case and matched samples.

One-to-one greedy nearest-neighbor matching was performed without replacement in the original order of treated patients. Each plasma recipient was matched to the unused control patient with the closest logit propensity score, provided that the absolute logit propensity score difference was within a caliper width of 0.2 × standard deviation (SD) of the logit propensity score (caliper = 0.294; SD of the logit propensity score = 1.47 in the analytic dataset).

Missing data were handled as follows. Among the eight propensity score matching variables, the overall missingness was: age (2/156, 1.3%), sex (1/156, 0.6%), PaO2/FiO2 ratio (25/156, 16.0%), hemoglobin (1/156, 0.6%), platelet count (1/156, 0.6%), APTT (2/156, 1.3%), invasive ventilation status (1/156, 0.6%), and recorded comorbidity count (1/156, 0.6%). Complete data for all PSM covariates and outcome were available for 131/156 patients (84.0%), and 25/156 patients (16.0%) were excluded from PSM candidacy because of incomplete PSM covariates and/or outcome. Excluded patients included 5 plasma recipients and 20 controls; the main driver was missing PaO2/FiO2 ratio. Complete-case versus excluded-patient summaries are reported in Additional file 6. COVID-19 infection status was missing in 25 patients (16.0%); these patients were excluded from the COVID-19 subgroup analysis but included in all analyses that did not require COVID-19 status.

Balance after matching was assessed using standardized mean differences (SMD). SMD values < 0.1 were considered negligible imbalance and values < 0.2 were considered acceptable residual imbalance in this small matched sample [15].

### Statistical analysis

#### Baseline comparisons

Before PSM, continuous variables were compared using the Mann–Whitney U test and presented as medians with interquartile ranges (IQR); categorical variables were compared using the chi-square test or Fisher’s exact test and presented as counts with percentages. After PSM, paired comparisons were performed using the Wilcoxon signed-rank test for continuous variables and McNemar’s test for categorical variables.

#### Survival analysis

Kaplan–Meier survival curves were constructed for both unmatched and matched cohorts, and survival differences were assessed using the log-rank test. The time origin was hospital admission, and the event was in-hospital death. Patients who survived to discharge or were transferred to another hospital were censored at the discharge or transfer date.

#### Cox proportional hazards regression

Four Cox proportional hazards models were fitted: Model 1, unadjusted (plasma transfusion only); Model 2, adjusted for age and sex; Model 3, fully adjusted for age, sex, PaO2/FiO2 ratio, hemoglobin, APTT, invasive ventilation, and comorbidity count; and Model 4, PSM-adjusted (fully adjusted model applied to the matched cohort). Model 4 was considered supportive because it included the same covariates in a small matched cohort and did not use additional matched-pair clustering. The proportional hazards assumption was assessed using Schoenfeld residuals. When global or covariate-specific proportional hazards assumptions were not met, Cox hazard ratios (HRs) were interpreted as time-weighted associations over follow-up rather than constant effects; the true time-varying effect could not be inferred from these Cox HRs alone.

#### Inverse probability of treatment weighting

As a supplement to PSM, inverse probability of treatment weighting (IPTW) was applied to the complete-case cohort (n = 131) using the same propensity score model. Stabilized average treatment effect (ATE) weights were computed as: treated patients, P̂(T = 1)/ê; control patients, P̂(T = 0)/(1 − ê), where P̂(T = 1) = 57/131 and ê is the individual propensity score. Overlap weights (treated: 1 − ê; control: ê) were also computed as a sensitivity analysis that emphasizes patients in the propensity score overlap region and achieved excellent covariate balance in this dataset (all SMD < 0.1). Weighted Cox models were fitted using sandwich variance. Weighted balance statistics were rounded to three decimal places.

#### Exploratory laboratory-respiratory severity index

Because SOFA score was unavailable for all patients, an exploratory laboratory-respiratory severity index was constructed via principal component analysis (PCA) as a data-driven sensitivity probe for disease severity. Fifteen variables with > 84% completeness were included: 12 standard laboratory values (white blood cell count [WBC], neutrophil count, lymphocyte count, hemoglobin, platelet count, CRP, ALT, AST, creatinine, PT, APTT, and fibrinogen), PaO₂/FiO₂ ratio, invasive ventilation status, and age. The PCA was performed as a complete-case analysis among 130 patients with non-missing values for all 15 variables. Continuous variables were standardized to mean 0 and standard deviation (SD) 1 before PCA. The first principal component (PC1) was extracted and oriented so that higher values correspond to greater severity (positive correlation with mortality). PC1 explained 18.5% of total variance. This index was used only in a standalone sensitivity Cox model (plasma + age + sex + severity index + comorbidity count) and was not added to the primary full model, which already includes several of the variables contributing to PC1. Age was retained as an individual covariate in this sensitivity Cox model for clinical interpretability; the PC1 loading for age was small.

#### Competing risk analysis

Because discharge or transfer alive removes patients from the risk set for in-hospital death, a Fine-Gray subdistribution hazard model was fitted as a sensitivity analysis, treating discharge or transfer alive as a competing event rather than right-censoring it. The Fine-Gray subdistribution hazard ratio (SHR) reflects the effect of plasma transfusion on the cumulative incidence of in-hospital death, accounting for the competing risk of discharge or transfer alive. The same covariates as the primary fully adjusted model (age, sex, PaO₂/FiO₂ ratio, hemoglobin, APTT, invasive ventilation, comorbidity count) were included. The SHR and Cox cause-specific HR are complementary estimands: the SHR pertains to cumulative incidence, whereas the HR pertains to the instantaneous cause-specific hazard.

#### Subgroup analysis

Exploratory subgroup analyses were performed descriptively by age (< 65 vs. ≥ 65 years), sex, invasive ventilation status, and COVID-19 infection status. Formal interaction tests were not performed because several matched subgroup cells were sparse and the analyses were not powered for subgroup-treatment interaction.

#### Sensitivity analyses

Several sensitivity analyses were conducted: (1) alternative outcome definition (patients discharged against medical advice classified as survivors); (2) exploratory mortality analyses by plasma initiation onset day, total plasma volume, and duration of plasma use among plasma recipients; (3) exclusion of two plasma recipients identified during source-data review as possible plasma-exchange cases; (4) IPTW and overlap weighting using the same propensity score model; (5) a standalone sensitivity Cox model incorporating an exploratory laboratory-respiratory severity index derived from PCA; (6) Fine-Gray competing risk analysis treating discharge or transfer alive as a competing event; (7) E-value calculation; (8) full-cohort multivariable logistic regression using the same covariate set as the fully adjusted Cox model; (9) a coagulation-enhanced propensity score sensitivity analysis adding PT and fibrinogen to the primary propensity score covariates; (10) descriptive microbiological and coagulation-context summaries; and (11) exploratory time-dependent Cox models using recovered onset-day and verified-date timing information. These timing models were used to assess the potential direction and magnitude of time-dependent exposure bias but were not treated as definitive landmark or primary time-dependent models because admission-relative initiation time was not consistently reliable. Additional exploratory sensitivity analyses are summarized in Additional file 6.

All statistical analyses were performed using Python 3.12 (Python Software Foundation) with pandas 2.3.3, numpy 2.4.4, scipy 1.17.1, scikit-learn 1.8.0, lifelines 0.30.3, and matplotlib 3.10.8; the Fine-Gray competing risk analysis was performed using R 4.5.1 with the cmprsk package. A two-sided p-value < 0.05 was considered statistically significant.

## Results

### Study population

**Fig. 1:**
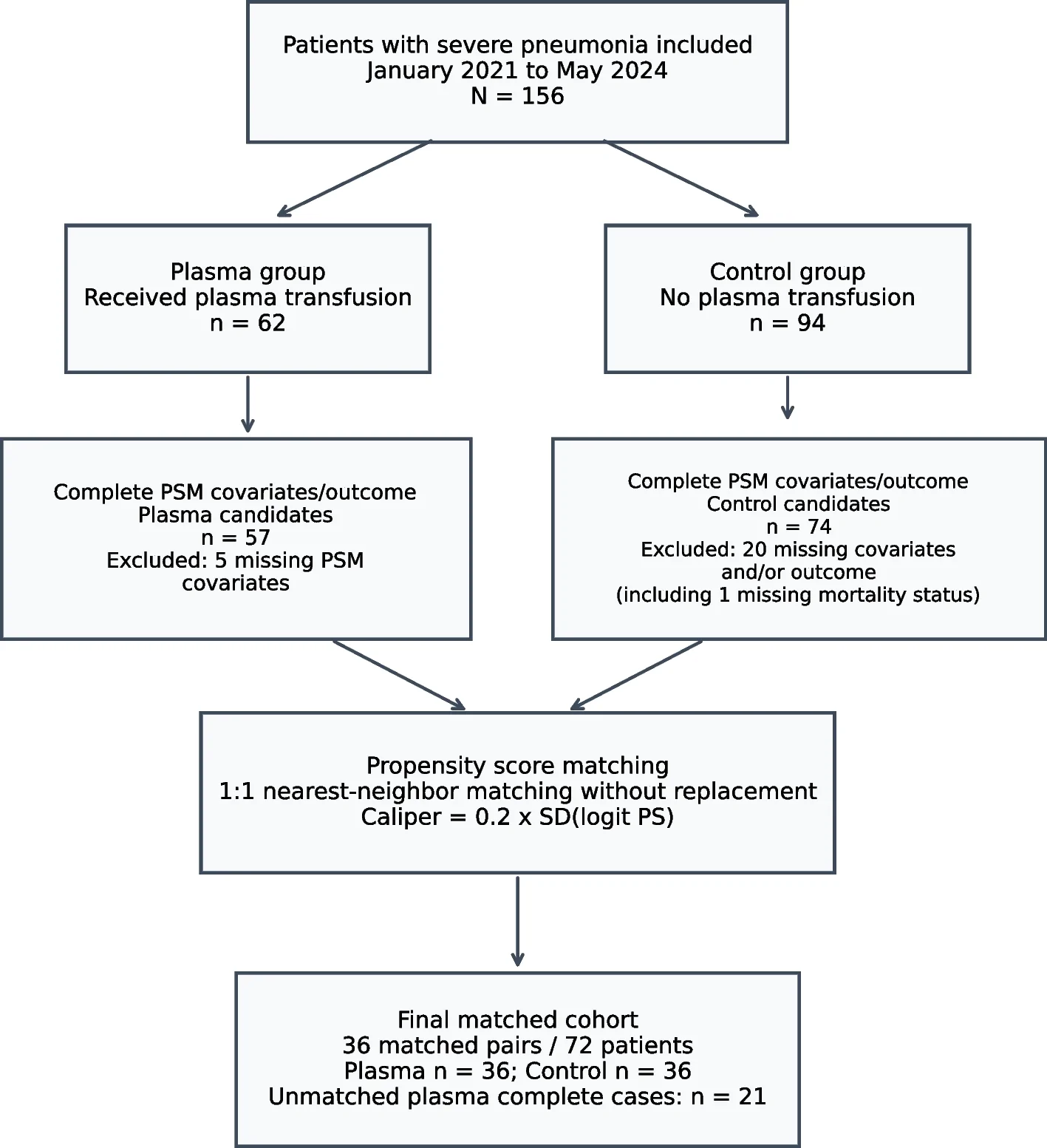
Study flow diagram showing patient selection and propensity score matching process. PSM = propensity score matching. The control exclusion count includes one patient with missing mortality status within the 20 records with missing covariates and/or outcome

A total of 156 patients with severe pneumonia were included in the study, comprising 62 patients who received plasma transfusion and 94 who did not (Fig. 1). Complete data for the propensity score covariates and outcome were available for 131 patients (57 plasma recipients and 74 controls), who entered the matching procedure. After propensity score matching, 36 matched pairs (72 patients) were formed. Of the original 62 plasma recipients, 5 were excluded because of incomplete matching covariates and 21 complete-case patients were not matched within the caliper, reflecting limited propensity-score overlap for some treated patients. The primary matched analysis therefore included 72 of the original 156 patients (46.2%). Among available severe-pneumonia phenotype fields, invasive mechanical ventilation was recorded in 93/155 patients (60.0%), non-invasive ventilation in 42/153 (27.5%), and PaO2/FiO2 < = 250 in 81/131 patients (61.8% with available PaO2/FiO2). Structured vasopressor use, minor-criteria counts, and pneumonia acquisition category were not available.

### Baseline characteristics before and after PSM

Before PSM, the plasma group had significantly worse baseline characteristics compared to controls (Table 1). The most notable imbalances were: invasive ventilation rate (82.3% vs. 45.2%, |SMD|= 0.83), hemoglobin (median 101.0 vs. 119.0 g/L, |SMD|= 0.74), platelet count (median 163.0 vs. 222.0 × 10⁹/L, |SMD|= 0.48), APTT (median 31.0 vs. 27.6 s, |SMD|= 0.52), and PaO₂/FiO₂ ratio (median 196.6 vs. 238.8, |SMD|= 0.41). These differences indicated substantial confounding by indication: patients receiving plasma were considerably sicker at baseline.

**Table 1:** Baseline characteristics before and after propensity score matching

Variable	Plasma before PSM (*n* = 62)	Control before PSM (*n* = 94)	SMD before PSM	Plasma after PSM (*n* = 36)	Control after PSM (*n* = 36)	SMD after PSM
Age, years	71.5 (63.2–81.0)	71.5 (63.8–76.8)	0.04	71.5 (64.8–81.0)	74.0 (64.8–79.0)	0.03
Male sex	46/62 (74.2%)	69/93 (74.2%)	0.00	24/36 (66.7%)	26/36 (72.2%)	0.12
PaO₂/FiO₂ ratio	196.6 (118.6–301.8)	238.8 (154.0–337.9)	0.41	216.4 (147.3–308.9)	188.9 (130.8–279.9)	0.02
Hemoglobin, g/L	101.0 (81.2–113.8)	119.0 (106.0–131.0)	0.74	107.0 (96.5–131.8)	113.5 (93.8–121.2)	0.03
Platelet count, × 10⁹/L	163.0 (101.8–253.8)	222.0 (168.0–301.0)	0.48	176.5 (148.5–262.2)	198.5 (137.5–275.5)	0.07
APTT, seconds	31.0 (26.7–37.6)	27.6 (25.8–31.8)	0.52	29.9 (26.4–34.0)	29.0 (26.2–36.1)	0.20
Invasive ventilation	51/62 (82.3%)	42/93 (45.2%)	0.83	26/36 (72.2%)	23/36 (63.9%)	0.18
Comorbidity count	2.0 (1.0–3.0)	2.0 (1.0–3.0)	0.04	2.0 (1.0–3.0)	2.0 (2.0–3.0)	0.11
In-hospital mortality	40/62 (64.5%)	26/93 (28.0%)	0.78	18/36 (50.0%)	13/36 (36.1%)	0.28

Values are median (IQR) or counts/available denominators (n/N) with percentages. SMD = standardized mean difference. Complete data for all PSM covariates and outcome were available for 131/156 patients (84.0%); 25 patients (16.0%; 5 plasma and 20 control) were excluded from PSM candidacy because of incomplete PSM covariates and/or outcome, mainly missing PaO2/FiO2 ratio. Complete-case versus excluded-patient summaries are provided in Additional file 6. Denominators may vary according to available data for each variable. One control record had missing sex, mortality status, and several PSM covariates; therefore, the unmatched control denominator was 93 for sex, invasive ventilation, and mortality. Comorbidity count refers to the pre-existing source-dataset aggregate count used as recorded in propensity-score estimation and adjusted models; its exact per-patient decomposition could not be fully reconstructed from available source documentation. DIC was recorded separately and was not entered as an independent model covariate because it was sparse

*APTT* activated partial thromboplastin time, *PaO2/FiO2* ratio of partial pressure of arterial oxygen to fraction of inspired oxygen

After PSM, measured baseline covariates showed improved balance by SMD (most |SMD|≤ 0.2; Table 1). Improvements in balance were observed for invasive ventilation (|SMD| from 0.83 to 0.18) and hemoglobin (|SMD| from 0.74 to 0.03).

### Primary outcome: in-hospital mortality

#### Unmatched cohort

In the unmatched cohort, mortality was significantly higher in the plasma group than in controls (64.5% vs. 28.0%, *p* < 0.001; crude odds ratio [OR] 4.69, 95% confidence interval [CI] 2.35–9.34). The control-group mortality denominator was 93 because one control patient had missing mortality status.

#### Matched cohort

After PSM, the mortality difference was substantially attenuated: 50.0% in the plasma group vs. 36.1% in the control group. McNemar's test yielded a *p*-value of 0.383, indicating no statistically significant difference. The matched OR was 1.62 (95% CI 0.67–3.92), indicating that plasma recipients had approximately 1.6-fold higher odds of death, but the confidence interval included unity. The non-significant result should not be interpreted as evidence of no effect, because the confidence interval is compatible with both a lower and a substantially higher odds of mortality.

As a post-hoc precision check, the 21 discordant matched pairs provide limited power for moderate paired effects. Conditional on 21 discordant pairs, an exact two-sided McNemar/binomial test at alpha = 0.05 would have approximately 25% power to detect a paired OR of 2.0, 57% power for an OR of 3.0, and 77% power for an OR of 4.0. The non-significant result therefore reflects imprecision and should not be read as evidence that plasma transfusion has no association with mortality.

The paired outcome distribution in the matched cohort showed 5 pairs where both patients died, 13 pairs where only the plasma patient died, 8 pairs where only the control patient died, and 10 pairs where both survived.

#### Cox proportional hazards regression

The results of the four Cox regression models are presented in Table 2. The HR for plasma transfusion showed substantial attenuation with increasing adjustment: unadjusted HR 1.55 (95% CI 0.93–2.59, *p* = 0.093); age- and sex-adjusted HR 1.57 (95% CI 0.94–2.64, *p* = 0.087); fully adjusted HR 0.85 (95% CI 0.43–1.67, *p* = 0.634); and PSM-adjusted HR 0.61 (95% CI 0.27–1.37, *p* = 0.232). The direction reversal between the matched-pair McNemar OR (1.62) and the PSM-adjusted Cox HR (0.61) reflects different estimands and time structures: the OR summarizes binary mortality in matched pairs, whereas the HR incorporates event timing and censoring from hospital admission. Because plasma was classified as an any-time exposure, recipients also had to survive until transfusion, making time-independent Cox HRs vulnerable to immortal-time bias. Visual inspection of the matched Kaplan–Meier curves suggested possible time-varying effects; therefore, Cox estimates should be interpreted as time-weighted associations over follow-up, and the true time-varying effect cannot be inferred from these HRs alone. Schoenfeld diagnostics indicated global non-proportionality in the fully adjusted unmatched model (Model 3; global *p* = 0.0005; plasma variable *p* = 0.694), whereas Model 4 did not reject the proportional hazards assumption globally (global *p* = 0.169; plasma variable *p* = 0.582), although the comorbidity count covariate showed evidence of non-proportionality (*p* = 0.028). Cox HRs below 1.0 should therefore not be interpreted as evidence of benefit.

**Table 2:** Cox proportional hazards regression models for in-hospital mortality

Variable	M1 HR (95% CI)	M1 p	M2 HR (95% CI)	M2 p	M3 HR (95% CI)	M3 p	M4 HR (95% CI)	M4 p
Plasma transfusion	1.55 (0.93–2.59)	0.093	1.57 (0.94–2.64)	0.087	0.85 (0.43–1.67)	0.634	0.61 (0.27–1.37)	0.232
Age, per year	NI	NI	1.01 (0.99–1.03)	0.236	1.02 (1.00–1.05)	0.063	1.04 (1.00–1.07)	0.035
Female sex	NI	NI	0.54 (0.27–1.06)	0.074	0.59 (0.29–1.21)	0.149	0.58 (0.22–1.52)	0.272
PaO₂/FiO₂, per 50-unit increase	NI	NI	NI	NI	0.85 (0.74–0.98)	0.028	0.81 (0.67–0.99)	0.041
Hemoglobin, per g/L	NI	NI	NI	NI	0.98 (0.96–0.99)	0.002	0.99 (0.97–1.02)	0.528
APTT, per second	NI	NI	NI	NI	1.01 (0.99–1.03)	0.424	1.02 (0.96–1.10)	0.513
Invasive ventilation	NI	NI	NI	NI	4.01 (1.39–11.58)	0.010	3.11 (0.92–10.53)	0.068
Comorbidities, per unit	NI	NI	NI	NI	0.85 (0.68–1.06)	0.144	0.97 (0.71–1.32)	0.825

Model 1 included plasma transfusion only. Model 2 adjusted for age and sex. Model 3 was fully adjusted for age, sex, PaO2/FiO2 ratio, hemoglobin, APTT, invasive ventilation, and recorded comorbidity count. The recorded comorbidity count refers to the same pre-existing source-dataset aggregate described in the Methods and Table 1 note. Model 4 used the propensity score-matched cohort with the same covariates as Model 3. Model sample sizes were 155, 154, 131, and 72 patients, respectively. PaO2/FiO2 ratio was rescaled per 50-unit increase for interpretability. The fully adjusted unmatched model violated the global proportional hazards assumption; Cox HRs should be interpreted as time-weighted associations rather than constant effects

*HR* hazard ratio, *CI* confidence interval, *NI* not included in the corresponding model

The crude OR and unadjusted Cox HR also differed in statistical significance because they summarize different aspects of the same exposure-outcome relationship: the OR compares cumulative in-hospital mortality proportions, whereas the Cox model incorporates event timing and censoring from hospital admission. These estimates should therefore be interpreted as complementary rather than interchangeable summaries.

In the fully adjusted model (Model 3), invasive ventilation was the strongest predictor of mortality (HR 4.01, 95% CI 1.39–11.6, *p* = 0.010), followed by hemoglobin (HR 0.98 per g/L, 95% CI 0.96–0.99, *p* = 0.002) and PaO₂/FiO₂ ratio (HR 0.85 per 50-unit increase, 95% CI 0.74–0.98, *p* = 0.028). In the PSM-adjusted model (Model 4), age was associated with higher mortality (HR 1.04 per year, 95% CI 1.00–1.07, *p* = 0.035), while invasive ventilation was associated with a higher point estimate for mortality that did not reach statistical significance (HR 3.11, 95% CI 0.92–10.53, *p* = 0.068).

#### Kaplan–Meier survival analysis

**Fig. 2:**
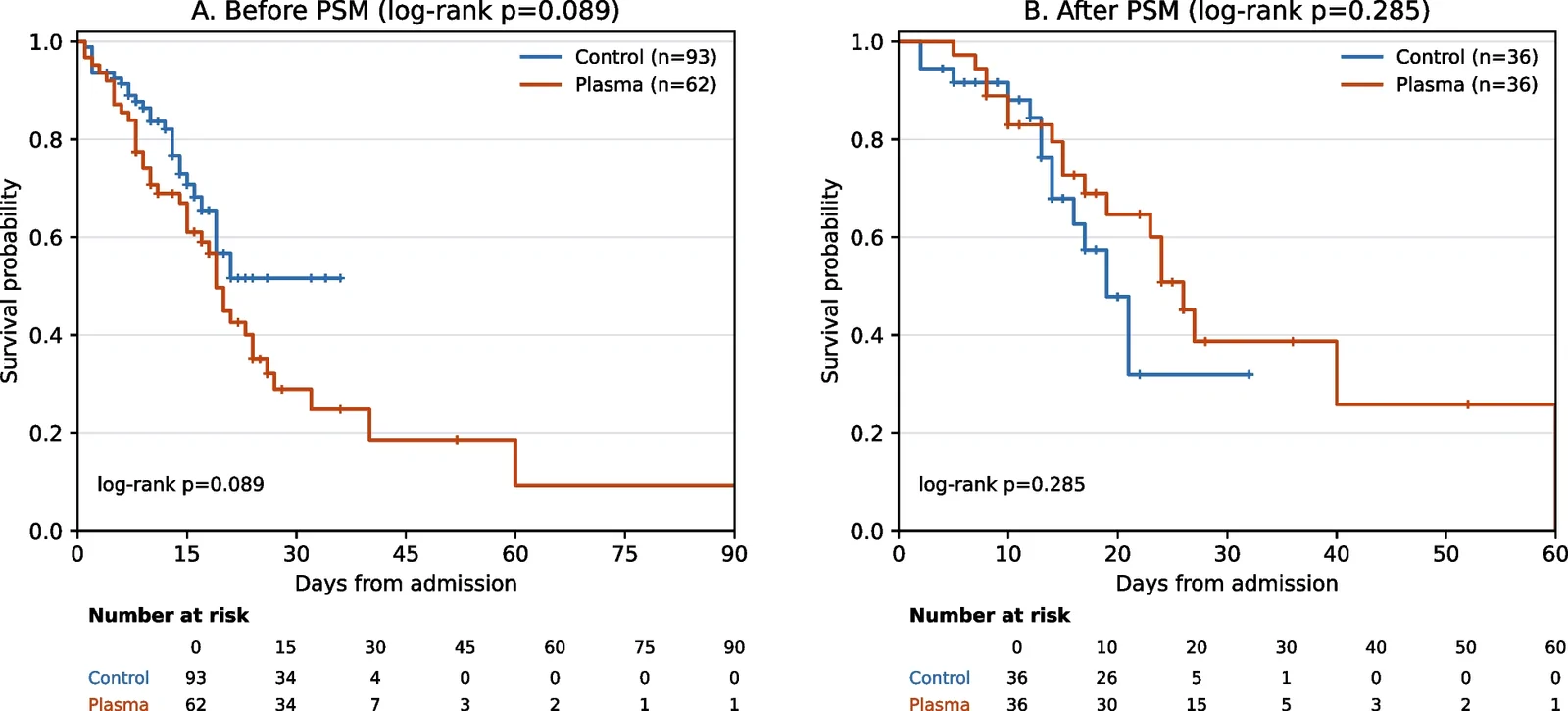
Kaplan–Meier survival curves for in-hospital mortality with number-at-risk tables. **A** Outcome-available unmatched cohort (log-rank *p* = 0.089), displayed through 90 days for readability; the median survival time was not reached for controls (lower 95% CI 19.0 days) and was 19.0 days (95% CI 15.0–24.0) for plasma recipients. **B** Propensity score-matched cohort (log-rank *p* = 0.285), displayed through 60 days; median survival was 19.0 days (95% CI 14.0-not reached) for controls and 26.0 days (95% CI 17.0–60.0) for plasma recipients. Crossing of the matched curves is consistent with possible time-varying effects and supports cautious interpretation of Cox HRs

Before PSM, the Kaplan–Meier survival curves suggested poorer survival for the plasma group, but the difference did not reach statistical significance (log-rank *p* = 0.089; Fig. 2A). The median survival time was 19.0 days for the plasma group and was not reached for the control group within the observed control follow-up. The 30-day survival probabilities were 28.9% and 51.6%, respectively.

After PSM, the survival difference was reduced and remained statistically non-significant (log-rank *p* = 0.285; Fig. 2B). The median survival time was 26.0 days (95% CI 17.0–60.0) for the plasma group and 19.0 days (95% CI 14.0-not reached) for the control group. The matched curves crossed, supporting caution in interpreting a single proportional-hazards estimate. The 30-day survival probabilities were 38.7% and 31.9%, respectively.

### Secondary outcome: hospital length of stay

Hospital length of stay was longer among plasma recipients before PSM (median 15.5 days, IQR 8.2–23.8) than among controls (median 12.0 days, IQR 8.0–17.0; Mann–Whitney U *p* = 0.037). After PSM, length of stay remained longer in the plasma group (median 17.0 days, IQR 10.8–25.2) than in matched controls (median 13.5 days, IQR 8.8–17.0; Wilcoxon signed-rank *p* < 0.001).

#### Subgroup analysis

**Fig. 3:**
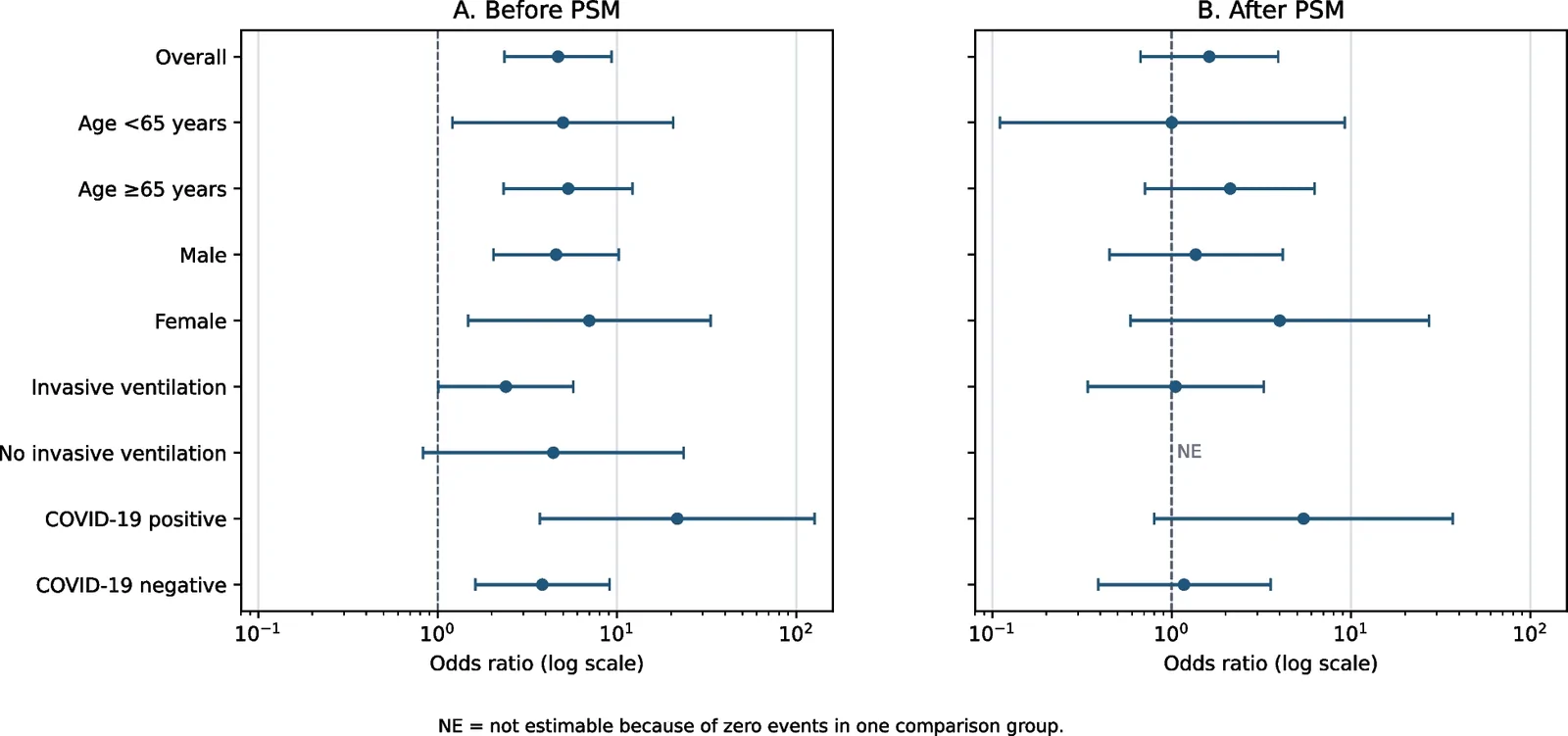
Forest plot of exploratory subgroup analyses for the association between plasma transfusion and mortality. OR = odds ratio; CI = confidence interval; NE = not estimable because of zero events in one comparison group. After-PSM subgroup estimates are descriptive 2 × 2 estimates rather than matched-pair estimates

Exploratory subgroup analyses were performed in both the unmatched and matched cohorts (Fig. 3, Table 3). Because subgroup analyses were not adjusted for multiplicity and several matched subgroups were small, these results should be interpreted descriptively.

**Table 3:** Exploratory subgroup analysis of the association between plasma transfusion and mortality

Cohort	Subgroup	Plasma mortality	Control mortality	OR (95% CI)	*p* value
Before PSM	Overall	40/62 (64.5%)	26/93 (28.0%)	4.69 (2.35–9.34)	< 0.001
Before PSM	Age < 65 years	9/18 (50.0%)	4/24 (16.7%)	5.00 (1.21–20.61)	0.026
Before PSM	Age ≥ 65 years	31/44 (70.5%)	21/68 (30.9%)	5.34 (2.33–12.20)	< 0.001
Before PSM	Male	32/46 (69.6%)	23/69 (33.3%)	4.57 (2.05–10.21)	< 0.001
Before PSM	Female	8/16 (50.0%)	3/24 (12.5%)	7.00 (1.48–33.21)	0.014
Before PSM	Invasive ventilation	37/51 (72.5%)	22/42 (52.4%)	2.40 (1.01–5.70)	0.047
Before PSM	No invasive ventilation	3/11 (27.3%)	4/51 (7.8%)	4.41 (0.83–23.50)	0.083
Before PSM	COVID-19 positive	13/16 (81.2%)	3/18 (16.7%)	21.67 (3.71–126.48)	< 0.001
Before PSM	COVID-19 negative	23/41 (56.1%)	14/56 (25.0%)	3.83 (1.62–9.09)	0.002
After PSM	Overall	18/36 (50.0%)	13/36 (36.1%)	1.62 (0.67–3.92)	0.383
After PSM	Age < 65 years	2/9 (22.2%)	2/9 (22.2%)	1.00 (0.11–9.23)	1.000
After PSM	Age ≥ 65 years	16/27 (59.3%)	11/27 (40.7%)	2.12 (0.71–6.27)	0.176
After PSM	Male	12/24 (50.0%)	11/26 (42.3%)	1.36 (0.45–4.16)	0.586
After PSM	Female	6/12 (50.0%)	2/10 (20.0%)	4.00 (0.59–27.25)	0.157
After PSM	Invasive ventilation	15/26 (57.7%)	13/23 (56.5%)	1.05 (0.34–3.26)	0.934
After PSM	No invasive ventilation	3/10 (30.0%)	0/13 (0.0%)	NE	NE
After PSM	COVID-19 positive	7/10 (70.0%)	3/10 (30.0%)	5.44 (0.80–36.87)	0.082
After PSM	COVID-19 negative	11/26 (42.3%)	10/26 (38.5%)	1.17 (0.39–3.56)	0.778

The After-PSM Overall row reports the paired McNemar OR and exact McNemar p value. Subgroup ORs are unpaired descriptive 2 × 2 estimates because matched-pair estimators were not stable in small subgroup cells. *P* values < 0.001 are not reported to greater precision. Subgroup analyses were exploratory and were not adjusted for multiplicity

*OR* odds ratio, *CI* confidence interval, *PSM* propensity score matching, *NE* not estimable because of zero events in one comparison group

In the unmatched cohort, the association between plasma transfusion and mortality was present across most subgroups but appeared strongest among COVID-19 positive patients (OR 21.67, 95% CI 3.71–126.48, *p* < 0.001), compared with COVID-19 negative patients (OR 3.83, 95% CI 1.62–9.09, *p* = 0.002). Among patients requiring invasive ventilation, the OR was 2.40 (95% CI 1.01–5.70, *p* = 0.047), while among those without invasive ventilation, the OR was 4.41 (95% CI 0.83–23.50, *p* = 0.083). The association was directionally similar across age and sex subgroups.

In the PSM-matched cohort, subgroup estimates were attenuated and imprecise. The COVID-19 subgroup analysis was limited by small sample size (*n* = 20 in the COVID-positive subgroup after matching), and the OR was 5.44 (95% CI 0.80–36.87,* p* = 0.082), with a wide confidence interval including unity. Among patients with invasive ventilation (*n* = 49 after matching), the OR was 1.05 (95% CI 0.34–3.26, *p* = 0.93). In the matched subgroup without invasive ventilation, the control group had no deaths, so the OR was not estimable. These exploratory findings should not be interpreted as evidence of subgroup-specific treatment effects.

#### Sensitivity analyses

##### Alternative outcome definition

When patients discharged against medical advice were classified as survivors rather than deaths, mortality was 13/62 (21.0%) in the plasma group and 6/93 (6.5%) in controls. The crude OR decreased from 4.69 to 3.85 (95% CI 1.38–10.76, Fisher exact *p* = 0.011), but the direction of the association remained unchanged.

##### Plasma initiation timing

Among the 62 plasma recipients, the median onset day of plasma initiation was day 4.0 (IQR 2.0–10.0). Initiation timing did not differ significantly between patients who died and survived (median day 3.5 vs. 4.5; Mann–Whitney *p* = 0.277). When analyzed as a continuous variable, each one-day later onset-day initiation was not significantly associated with mortality (OR 0.96, 95% CI 0.89–1.04, *p* = 0.350). Early-versus-late exploratory cutoffs also showed no statistically significant association with mortality (≤ day 2 vs. > day 2, Fisher *p* = 0.262; ≤ day 3 vs. > day 3, *p* = 0.424; ≤ day 7 vs. > day 7, *p* = 1.000; Additional file 2: Supplementary Table S2). Because the timing field reflected onset-day rather than consistently verified admission-relative exposure time, these analyses were interpreted descriptively.

##### Plasma volume and duration analyses

Among 60 of 62 plasma recipients with available volume data, patients were divided into rank-based tertiles by total plasma volume: low volume (200–600 mL, *n* = 24), medium volume (650–1150 mL, *n* = 16), and high volume (1300–6000 mL, *n* = 20). Mortality rates were 58.3%, 56.2%, and 75.0%, respectively, with no statistically significant difference across volume strata (chi-square *p* = 0.41; Spearman *ρ* = 0.16, *p* = 0.23). Duration of plasma use was available for all 62 plasma recipients. Mortality was 61.9% for short duration (1–2 days), 65.0% for medium duration (2–5 days), and 66.7% for long duration (6–20 days), with no significant difference across duration strata (chi-square *p* = 0.948; Spearman *ρ* = 0.02, *p* = 0.866). These analyses are summarized in Table 4. Because treatment duration depends partly on survival time, duration analyses were interpreted descriptively rather than causally.

**Table 4:** Exploratory mortality analyses by plasma volume and duration among plasma recipients

Analysis	Group	Range	n	Deaths	Mortality	Overall p	Trend p
Total plasma volume	Low	200–600 mL	24	14	58.3%	0.410	0.230
Total plasma volume	Medium	650–1150 mL	16	9	56.2%		
Total plasma volume	High	1300–6000 mL	20	15	75.0%		
Plasma-use duration	Short	1–2 days	21	13	61.9%	0.948	0.866
Plasma-use duration	Medium	2–5 days	20	13	65.0%		
Plasma-use duration	Long	6–20 days	21	14	66.7%		

Volume groups were defined by rank-based tertiles among the 60 plasma recipients with available volume data; group sizes were unequal because volume values clustered at common transfusion amounts around the tertile boundaries. Duration groups were defined by rank-based tertiles among all 62 plasma recipients with available duration data. Overall p values are chi-square p values comparing mortality across strata. Trend *p* values are Spearman rank-correlation *p* values. These descriptive analyses did not demonstrate a statistically significant exposure-intensity gradient. Duration analyses should not be interpreted causally because treatment duration is susceptible to survivor bias

##### Possible plasma-exchange cases

When two plasma recipients identified during source-data review as possible plasma-exchange cases were excluded, the crude mortality association remained (38/60 [63.3%] vs. 26/93 [28.0%]; OR 4.45, 95% CI 2.22–8.90, *p* < 0.001). After refitting the propensity score and rematching, 35 matched pairs were formed; the matched McNemar OR was 1.17 (*p* = 1.000), and the PSM-adjusted Cox HR for plasma transfusion was 0.44 (95% CI 0.20–1.01, *p* = 0.052). This sensitivity analysis did not support an independent harmful association after adjustment.

E-value for unmeasured confounding: The E-value for the crude OR (4.69) was 8.84, and the E-value for the fully adjusted HR (0.85) was 1.63 [16]. These values indicate that an unmeasured confounder would need to have a risk ratio of at least 1.63 with both plasma transfusion and mortality to fully explain the observed association after adjustment. This threshold is modest and does not rule out residual confounding, particularly given the absence of SOFA score and transfusion indication data.

##### Exploratory laboratory-respiratory severity index

To probe the effect of severity adjustment in the absence of SOFA score, an exploratory severity index was derived from the first principal component of 15 clinical variables (12 laboratory values + PaO₂/FiO₂ + invasive ventilation + age; Additional file 4: Supplementary Table S4). PC1 explained 18.5% of total variance and was significantly associated with mortality (point-biserial *r* = 0.288, *p* = 0.0009). In a standalone sensitivity Cox model adjusting for the severity index alongside age, sex, and comorbidity count, the plasma transfusion HR was 1.82 (95% CI 0.99–3.32, *p* = 0.053), compared with 2.01 (95% CI 1.10–3.66, *p* = 0.022) in the same model without the severity index. The severity index itself was a significant predictor (HR 1.19 per SD increase, 95% CI 1.05–1.35, *p* = 0.006). Adding the severity index attenuated the plasma association from statistically significant to borderline non-significant, but the point estimate remained above unity. In contrast, the fully adjusted model with individual laboratory variables (Model 3) yielded a plasma HR of 0.85 (95% CI 0.43–1.67, *p* = 0.634). This gradient — from HR 2.01 without severity adjustment to HR 1.82 with the composite index to HR 0.85 with individual lab variables — shows a pattern consistent with attenuation after more detailed adjustment for measured severity markers.

##### Competing risk analysis

Because discharge or transfer alive removes patients from the risk set for in-hospital death, a Fine-Gray subdistribution hazard model was fitted treating discharge or transfer alive as a competing event (Additional file 5: Supplementary Table S5). Among the 131 complete-case patients, 53 (40.5%) died and 78 (59.5%) were discharged or transferred alive; there were no patients with traditional right-censoring. The unadjusted SHR for plasma transfusion was 3.56 (95% CI 2.00–6.34, *p* < 0.001), which was larger than the unadjusted Cox HR of 2.03 (95% CI 1.12–3.67, *p* = 0.020) computed on the same *n* = 131 subset. This divergence arises because the Fine-Gray model directly estimates the cumulative incidence difference between groups, and differential discharge or transfer patterns — lower-risk control patients leave the hospital alive earlier — yield a larger cumulative-incidence contrast than the cause-specific hazard ratio. After full adjustment with the same covariates as primary Model 3, the SHR was 1.38 (95% CI 0.72–2.65, *p* = 0.329), which was not statistically significant, consistent with the fully adjusted Cox HR of 0.85 (95% CI 0.43–1.67, *p* = 0.634). The different point estimates reflect different estimands — the SHR pertains to cumulative incidence, whereas the Cox HR pertains to cause-specific hazard — rather than a contradictory finding. Invasive ventilation remained the strongest predictor in the Fine-Gray model (SHR 6.35, 95% CI 2.14–18.83, *p* = 0.001).

Additional exploratory sensitivity analyses: These analyses are summarized in Additional file 6 and Table 5. Full-cohort multivariable logistic regression was performed among 131 complete-case patients with 53 deaths using the same covariate set as the fully adjusted Cox model. In this binary outcome model, plasma transfusion was associated with higher odds of in-hospital mortality (adjusted OR 3.19, 95% CI 1.12–9.11, *p* = 0.030). Because this model does not incorporate exposure timing or censoring, it was interpreted as a complementary estimand rather than a replacement for the primary matched analysis. Like the time-to-event analyses, this binary any-transfusion exposure definition remains susceptible to exposure-timing misclassification and should not be interpreted as a definitive causal effect.

**Table 5:** Summary of primary and exploratory sensitivity estimates for plasma transfusion and in-hospital mortality

Analysis	Estimate for plasma	Direction	*p* value	Role and principal limitation
Primary matched McNemar analysis	OR 1.62 (95% CI 0.67–3.92)	Above 1.0; not significant	0.383	Primary binary matched estimand; small 36-pair cohort and limited overlap
Fully adjusted Cox model (Model 3)	HR 0.85 (95% CI 0.43–1.67)	Below 1.0; not significant	0.634	Supportive time-to-event model; global PH violated (*p* = 0.0005); not evidence of benefit
PSM-adjusted Cox model	HR 0.61 (95% CI 0.27–1.37)	Below 1.0; not significant	0.232	Supportive matched time-to-event model; exposure-timing and PH limitations; not evidence of benefit
Full-cohort logistic regression	Adjusted OR 3.19 (95% CI 1.12–9.11)	Above 1.0; significant	0.030	Exploratory cumulative-mortality estimand; ignores timing/censoring
Coagulation-enhanced PSM	OR 1.50 (95% CI 0.53–4.21)	Above 1.0; not significant	0.607	Exploratory PS model adding PT and fibrinogen; 34 pairs
Approximate adjusted time-dependent Cox	HR 1.73 (95% CI 0.85–3.51)	Above 1.0; not significant	0.132	Exploratory onset-day timing model; timing reliability limited
Strict verified-date adjusted time-dependent Cox	HR 2.67 (95% CI 1.38–5.16)	Above 1.0; significant	0.004	Exploratory verified-date model; selected records with valid dates

The matched McNemar analysis is the primary analysis. Other estimates are supportive or exploratory and differ by estimand, timing assumptions, model structure, and case selection. Cox HRs below 1.0 should not be interpreted as evidence of benefit

*CI* confidence interval, *HR* hazard ratio, *OR* odds ratio, *PH* proportional hazards, *PSM* propensity score matching

A coagulation-enhanced propensity score sensitivity analysis added PT and fibrinogen to the original propensity score variables. This analysis included 130 complete-case patients and formed 34 matched pairs. In the coagulation-enhanced matched cohort, mortality was 17/34 among plasma recipients and 14/34 among controls, yielding a matched OR of 1.50 (95% CI 0.53–4.21; exact McNemar *p* = 0.607). DIC was not forced into this model because DIC was sparse (3/156 patients, all in the plasma group), raising concern for unstable estimation; it was therefore reported descriptively.

Microbiological and coagulation context was summarized descriptively. Blood culture records, respiratory pathogen/culture records, Gram-negative organisms recorded in free-text fields, mixed organism categories, longer APTT, lower fibrinogen, and lower platelet count were more common among plasma recipients. These findings support the presence of clinically important baseline severity and indication differences, but they were descriptive only because pathogen fields were free text and sepsis-induced coagulopathy could not be defined without SOFA and INR.

Exploratory timing sensitivity outputs showed that recovered onset-day timing was available for all 62 plasma recipients, but exact admission-relative initiation time was not consistently reliable. Approximate time-dependent Cox models using source onset-day timing yielded HR 2.72 (95% CI 1.59–4.67, *p* < 0.001) in the unadjusted model and HR 1.73 (95% CI 0.85–3.51, *p* = 0.132) after adjustment. Strict verified-date time-dependent Cox models yielded HR 2.94 (95% CI 1.71–5.05, *p* < 0.001) in the unadjusted model and HR 2.67 (95% CI 1.38–5.16, *p* = 0.004) after adjustment. The strict verified-date adjusted model was limited to patients with reliably verifiable admission-relative dates and complete adjustment covariates (125 patients, 49 deaths). These exploratory estimates differed from the time-independent Cox HRs, reinforcing that time-to-event results are sensitive to exposure-timing assumptions and should not be interpreted as evidence of benefit. They were not treated as definitive correction for immortal-time bias because timing data quality and record selection remained important limitations.

## Discussion

This study examined a common clinical dilemma in severe pneumonia: plasma recipients had substantially higher crude mortality than non-recipients, but they also appeared substantially sicker at baseline. After PSM, the crude OR of 4.69 was attenuated to a matched OR of 1.62 (95% CI 0.67–3.92). This attenuation is consistent with confounding by indication accounting for a substantial component of the crude association. It does not prove that matching resolved confounding, and the matched estimate remained imprecise and compatible with clinically meaningful harm or no association. Cox estimates below unity after adjustment should not be interpreted as evidence of benefit, because the fully adjusted Cox model violated the global proportional hazards assumption and the exposure definition was vulnerable to immortal-time and time-dependent exposure bias.

For the clinician at the bedside, the practical implication is that a higher mortality rate among plasma recipients should not, on its own, lead to the conclusion that plasma caused the deaths. The patients who received plasma in this cohort were substantially sicker at baseline, with lower hemoglobin, lower platelet counts, prolonged APTT, lower fibrinogen, more microbiological documentation, and a much higher rate of invasive mechanical ventilation. These characteristics are closely related to the decision to order plasma and are also associated with mortality risk. When available measured confounders were balanced through matching, the mortality difference narrowed considerably. The key clinical message is one of interpretive caution: unadjusted comparisons between plasma recipients and non-recipients are likely confounded by the severity of illness and by the clinical indications that triggered transfusion.

The findings are consistent with prior evidence in several respects. A randomized trial found no reduction in bleeding complications from prophylactic plasma transfusion before invasive procedures in critically ill patients with coagulopathy [8], while observational and systematic-review evidence has linked plasma transfusion with adverse outcomes such as infection and acute lung injury in selected critically ill populations [17, 18]. The present study does not provide evidence that plasma transfusion improves survival in severe pneumonia. At the same time, because structured transfusion-indication data were unavailable, it also should not be used to make definitive judgments about the appropriateness of specific transfusion decisions in individual patients.

The COVID-19 subgroup showed a large crude association, but this estimate was markedly attenuated after matching and remained imprecise. This large unadjusted estimate may reflect especially strong confounding by indication during the COVID-19 period, when plasma-based therapies were more likely to be considered for patients with severe disease. Given the small matched COVID-positive subgroup, 16.0% missingness in COVID-19 status, and lack of multiplicity adjustment, this finding should be considered exploratory. COVID-19 and non-COVID-19 pneumonia may also differ in coagulopathy, inflammatory biology, and transfusion decision-making. Although RECOVERY and REMAP-CAP evaluated COVID-19 convalescent plasma rather than standard frozen plasma, their findings support caution in assuming mortality benefit from plasma-based interventions in severe respiratory illness [19, 20].

This study used PSM, Cox regression, subgroup analyses, and multiple sensitivity analyses to examine the stability and limitations of the findings. The estimates pointed in different directions: the primary matched OR (1.62), full-cohort logistic OR (3.19), coagulation-enhanced OR (1.50), and time-dependent HRs (1.73 and 2.67) were above unity, whereas the fully adjusted and PSM-adjusted Cox HRs (0.85 and 0.61) were below unity. This divergence reflects different estimands and time structures: logistic and matched ORs summarize cumulative mortality, Cox HRs weight event timing from admission, and time-dependent Cox models reclassify exposure over follow-up. The Cox HRs below unity most plausibly reflect proportional-hazards and timing limitations, including immortal-time bias, rather than a protective effect. These estimand-dependent findings reinforce caution rather than a single directional interpretation. The longer hospital stay among plasma recipients and the absence of a clear plasma-volume or duration-response gradient should also be interpreted descriptively, because length of stay, volume, and duration are influenced by survival time, discharge practice, indication, severity, and repeated exposure.

### Strengths

This study has several strengths that support its value as a transparent, hypothesis-generating analysis. It addresses a clinically important and under-studied question in severe pneumonia, a setting in which plasma is often used despite limited disease-specific evidence. It directly engages with confounding by indication rather than relying on unadjusted comparisons. It reports covariate balance before and after matching and triangulates the primary matched estimate with complementary sensitivity analyses, including weighting, competing-risk modeling, severity-index adjustment, E-value analysis, coagulation-enhanced propensity score matching, full-cohort logistic regression, and exploratory time-dependent Cox models. Finally, it explicitly reports key limitations and statistical assumptions, including missing SOFA score, absent transfusion-indication data, proportional-hazards violation, and immortal-time/time-dependent exposure bias.

Several limitations are important to consider. First, the retrospective single-center design, complete-case restriction, and small matched sample (36 pairs; 46.2% of the original cohort) limited precision and generalizability; the matched OR of 1.62 had a wide confidence interval (0.67–3.92) that does not rule out clinically meaningful harm or no association. Some complete-case plasma recipients could not be matched within the caliper; these unmatched complete-case plasma recipients had more severe baseline features, including invasive ventilation in 21/21, lower PaO2/FiO2, lower hemoglobin, lower platelet count, longer APTT, and mortality of 18/21 (85.7%), suggesting limited overlap for higher-propensity treated patients. Second, residual confounding remains a major concern because SOFA score was unavailable, INR was not available, and structured data on transfusion indication (active bleeding, procedural prophylaxis, laboratory abnormality without bleeding, DIC, or empirical supportive use during deterioration) were not consistently recorded. These scenarios may have very different baseline prognoses, so the absence of indication data limits the clinical meaning of any average effect. Platelet count and APTT were included in the primary propensity score, and PT and fibrinogen were added in the coagulation-enhanced sensitivity model as crude proxies for coagulopathy-related transfusion indication; however, these laboratory values remain imperfect proxies for the clinical decision to transfuse. Sepsis-induced coagulopathy could not be defined because SOFA and INR were unavailable, and DIC was too sparse for stable modeling. In addition, the source-dataset aggregate comorbidity count lacked a documented derivation rule, so its exact per-patient composition could not be reconstructed with complete certainty; sensitivity analysis using an explicit chronic/immune-status count with DIC excluded yielded a materially unchanged primary matched estimate. Third, plasma exposure was defined as any transfusion during hospitalization. Although onset-day timing was recovered from source records, exact admission-relative initiation time was not consistently reliable enough for a prespecified time-dependent or landmark primary analysis. Patients had to survive long enough to receive plasma, so immortal-time and time-dependent exposure biases may have biased time-to-event estimates in favor of the exposed group or attenuated harmful associations. Fourth, the fully adjusted unmatched Cox model violated the global proportional hazards assumption, further limiting interpretation of Cox HRs as constant effects over follow-up. Fifth, the available records did not consistently distinguish ordinary frozen plasma from fresh frozen plasma, and body weight was missing for most patients, precluding reliable product-subtype and mL/kg dose analyses. Sixth, severe pneumonia was defined using the 2007 IDSA/ATS severe pneumonia criteria, which were originally developed for community-acquired pneumonia; applying them to a broader severe-pneumonia cohort may limit phenotype specificity if hospital-acquired or ventilator-associated pneumonia cases were included. Finally, residual covariate imbalance after matching and the adjusted E-value of 1.63 indicate that residual unmeasured confounding remains a credible alternative explanation.

What should clinicians take from these findings for current practice? Plasma transfusion in severe pneumonia should continue to be guided by established hemostatic indications, such as active bleeding or clinically relevant coagulopathy before an invasive procedure [5, 21, 22]. When reviewing observational outcome data, clinicians should recognize that unadjusted comparisons between plasma recipients and non-recipients are highly vulnerable to confounding by indication. Although we could not directly evaluate the appropriateness of routine administration because explicit indication data were unavailable, we found no evidence of a survival benefit in this cohort; therefore, cautious use outside established hemostatic indications is warranted.

Moving forward, the key evidence gaps that need to be addressed through prospective research include: documentation of the specific clinical indication for each transfusion episode, categorized by bleeding, procedural prophylaxis, laboratory abnormality alone, or empirical supportive use; reliable recording of the time from admission to plasma initiation to support time-dependent analyses; collection of plasma product subtype and weight-based dosing where available; and inclusion of validated severity scores such as SOFA [23]. Studies designed with these elements would provide the granularity needed to determine which patients, if any, might benefit from plasma transfusion in the setting of severe pneumonia.

## Conclusions

In this retrospective cohort of patients with severe pneumonia, the crude association between plasma transfusion and higher mortality was attenuated after propensity score matching and adjustment for available measured confounders. The findings are consistent with confounding by indication accounting for a substantial component of the unadjusted mortality difference, but residual confounding cannot be excluded and the sensitivity analyses were estimand-dependent. Neither benefit nor harm could be determined because of residual confounding, small matched sample size, immortal-time/time-dependent exposure bias, violation of the proportional hazards assumption, and incomplete information on transfusion indication, product subtype, and validated severity scores. Prospective studies with structured collection of transfusion indication, timing, product subtype, weight-based dosing, and SOFA or similar severity measures are needed.

## Supplementary Information

Additional file 1: Supplementary Table S1. Laboratory characteristics in the complete-case cohort before propensity score matching; complete-case laboratory comparison between plasma recipients and controls.

Additional file 2: Supplementary Table S2. Plasma initiation timing sensitivity analyses, including exploratory analyses of plasma initiation onset day among plasma recipients.

Additional file 3: Supplementary Table S3. IPTW and overlap weighting sensitivity analyses, including covariate balance diagnostics, weighted Cox results, and weighting notes.

Additional file 4: Supplementary Table S4. Exploratory laboratory-respiratory severity index analysis, including PCA loadings and sensitivity Cox model results.

Additional file 5: Supplementary Table S5. Fine-Gray competing risk sensitivity analysis, including Cox and Fine-Gray estimates for in-hospital mortality with discharge or transfer alive treated as a competing event.

Additional file 6: Supplementary Table S6. Index and exploratory sensitivity analyses summarizing estimate direction, missingness/selection, matched versus unmatched plasma recipients, severe-pneumonia phenotype, power/precision, coagulation-enhanced PSM, full-cohort logistic regression, time-dependent Cox outputs, and microbiology/coagulation context.

## Data Availability

Aggregate data supporting the conclusions of this article are included within the article and its additional files. De-identified patient-level data are not publicly available because of institutional and ethical restrictions, but may be available from the corresponding author on reasonable request and subject to institutional approval.

## Abbreviations

ALT: Alanine aminotransferase
APTT: Activated partial thromboplastin time
AST: Aspartate aminotransferase
ATE: Average treatment effect
CI: Confidence interval
COVID-19: Coronavirus disease 2019
CRP: C-reactive protein
DIC: Disseminated intravascular coagulation
FFP: Fresh frozen plasma
HR: Hazard ratio
IDSA/ATS: Infectious Diseases Society of America/American Thoracic Society
IPTW: Inverse probability of treatment weighting
IQR: Interquartile range
OR: Odds ratio
PaO_2_/FiO_2_: Ratio of partial pressure of arterial oxygen to fraction of inspired oxygen
PCA: Principal component analysis
PC1: First principal component
PSM: Propensity score matching
PT: Prothrombin time
SD: Standard deviation
SHR: Subdistribution hazard ratio
SMD: Standardized mean difference
SOFA: Sequential Organ Failure Assessment
STROBE: Strengthening the Reporting of Observational Studies in Epidemiology
TRALI: Transfusion-related acute lung injury
WBC: White blood cell count
